# AGING ASSEMBLAGES: NEW MATERIALIST INSIGHTS IN THE SOCIOLOGY OF AGING

**DOI:** 10.1093/geroni/igae098.1973

**Published:** 2024-12-31

**Authors:** Vera Gallistl

**Affiliations:** Karl Landsteiner University of Health Sciences, Krems, Niederosterreich, Austria

## Abstract

In the last decades, scholars in gerontology and the sociology of aging have increasingly pursued overlapping ideas concerned with the materiality of aging. Central to these is the idea that studying materiality goes beyond a consideration of material objects, physical spaces, or aging bodies. Much rather, it has significant consequences for theory and methodology and hence offers a novel conceptual and empirical sociological lens for gerontology. In this paper, we put forward a new-materialist conceptualization of aging. Drawing on new materialist sociological theories, that have emerged in feminist sociology and the sociology of science and technology, we theorize aging not as an individual process, but as an aging assemblage that is distributed between humans, non-humans and more-than humans. We illustrate the fruitfulness of such a posthumanist understanding of aging by drawing on empirical examples from a qualitative case study on artificial intelligence in long-term care. We demonstrate how adopting a more-than-human perspective on aging offers unique pathways to understand how age-boundaries emerge through a complex interplay of actors and how these age-boundaries govern the unequal distribution of power and autonomy. By conceptualizing aging as an assemblage, we underscore the precarious nature of aging as a concept that is intertwined with complex systems of both situated difference and durable inequality, structural discrimination, and political and economic disadvantage. We conclude by discussing the implications of material gerontology for critical gerontology in an increasingly digitized and datafied world.

